# Observational, retrospective study of a large cohort of patients with Niemann-Pick disease type C in the Czech Republic: a surprisingly stable diagnostic rate spanning almost 40 years

**DOI:** 10.1186/s13023-014-0140-6

**Published:** 2014-09-19

**Authors:** Helena Jahnova, Lenka Dvorakova, Hana Vlaskova, Helena Hulkova, Helena Poupetova, Martin Hrebicek, Pavel Jesina

**Affiliations:** Institute of Inherited Metabolic Disorders, Charles University, First Faculty of Medicine, and General University Hospital in Prague, Prague, Czech Republic

**Keywords:** Niemann-Pick disease type C (NPC), Bone marrow smear (BMS), Cholesterol, Filipin, NPC1, NPC2, Diagnosis

## Abstract

**Background:**

Niemann-Pick disease type C (NPC) is a rare, fatal neurovisceral disorder with autosomal recessive inheritance, and featuring striking clinical variability dependent on the age at onset of neurological symptoms. We report data from a large cohort of 56 Czech patients with NPC diagnosed over a period of 37 years.

**Methods:**

An observational, retrospective analysis of historic and current clinical and laboratory information was performed among all NPC patients originating from the area of the contemporary Czech Republic and diagnosed between 1975 and 2012. All patients with ≥1 positive diagnostic test and relevant clinical information were included. Data on diagnostic methods (histopathological and/or ultrastructural; biochemical; genetic), clinical status and general information on treatment were collated. Data were examined in accordance with international guidelines for the management of NPC.

**Results:**

Between 1975 and 1985 diagnoses were based exclusively on specific histopathological findings, often at autopsy. Bone marrow smear (BMS) analyses have proved to be a very specific indicator for NPC and have become an important part of our diagnostic algorithm. Filipin staining and cholesterol esterification assays became the definitive diagnostic tests after 1985 and were applied in 24 of our patients. Since 2005, more and more patients have been assessed using *NPC1*/*NPC2* gene sequencing. Twelve patients were diagnosed with neonatal/early-infantile onset NPC, 13 with the late-infantile onset form, 20 with the juvenile onset form, and nine with the adolescent/adult onset form. Two diagnosed patients remained neurologically asymptomatic at study completion. Nineteen patients were siblings. Causal *NPC1* mutations were determined in 38 patients; two identical *NPC2* mutations were identified in one patient. In total, 30 different mutations were identified, 14 of which have been confirmed as novel. The frequency of individual mutated *NPC1* alleles in our cohort differs compared with previous published data: the most frequent mutant *NPC1* allele was p.R1186H (n = 13), followed by p.P1007A (n = 8), p.S954L (n = 8) and p.I1061T (n = 4).

**Conclusions:**

These data demonstrate the evolution of the diagnostic process in NPC over the last four decades. We estimate the contemporary birth prevalence of NPC in the Czech Republic at 0.93 per 100,000.

## Introduction

Niemann-Pick disease type C (NPC) is a rare genetically and clinically heterogeneous neurovisceral lysosomal storage disease associated with progressive, disabling neurological symptoms and premature death in most patients [1-3]. NPC usually occurs sporadically, and is inherited in an autosomal recessive fashion. It has been estimated to affect one case in every 100,000–120,000 live births [1,2,4,5].

The disease presents with striking variability. Most cases are recognised during childhood, but increasing numbers of patients are now being diagnosed with adolescent or adult onset of symptoms [6]. Data indicate that clinical symptomatology and disease progression in NPC are both markedly affected by the age at onset of neurological manifestations [2,7,8]. Patients are therefore categorized by age at onset of neurological manifestations based on early-infantile (<2 years), late-infantile (2 to <6 years), juvenile (6–15 years) and adolescent/adult-onset forms (>15 years) [8]. These categories are considered useful for the evaluation of disease course and responses to therapy [1].

Knowledge of the genetic causes, biochemical mechanisms, and natural history of the disease has helped greatly in improving diagnosis over the last two decades. However, the non-specific nature of many signs and symptoms, and the as yet incomplete characterization of underlying disease pathophysiology still represent significant hurdles to the timely detection and diagnosis of NPC [1,6,9]. Data from the largest international registry for NPC patients to date indicate marked delays between onset of initial neurological manifestations and diagnosis [10].

While expert consensus only distinguished NPC as a specific disorder separate from Niemann-Pick disease types A and B (NPA and NPB, which result from sphingomyelinase deficiency) in 1982 [11], experienced pathologists have been able to define NPC through complex histopathological analysis of key organs (especially brain, spleen and liver) at autopsy since the 1970s. Indeed, up to the late 1980s patients were exclusively diagnosed by means of histology, histochemistry and electron microscopy with a comprehensive assessment of all clinical data [12-15].

After definitive animal modelling studies in the mid-1980s, several research groups showed that NPC is a lipid trafficking disorder featuring dysregulation of intracellular lipid transport and metabolism [16-21]. As a result, the filipin staining test, which visualizes characteristic perinuclear accumulation of cholesterol-filled lysosomes in cultivated fibroblasts, with or without supporting evidence from cholesterol esterification assays, became regarded as the gold standard technique for diagnosing NPC [1,8]. Both methods are very specialized and labour-intensive. Similar to other diagnostic techniques in NPC, they also show some limitations, particularly in patients showing a ‘variant’ biochemical phenotype [1,8]. In addition, available data suggest that the filipin test has limited specificity [22].

The *NPC1* gene, which is associated with 95% of cases of NPC, was characterized in 1997 [23,24]; the *NPC2* gene was characterized soon after [25]. The number of NPC patients diagnosed based on genetic evidence of *NPC1* or *NPC2* gene mutations has increased markedly over the last decade due to methodological advances and improved suitability for pre-natal testing and genetic counselling [8]. Clinical scoring systems such as the NP-C Suspicion Index (SI) have also been developed to further accelerate detection and diagnosis [26].

All techniques used in the diagnostic process in NPC have their limits (Table 1). The diagnostic process for NPC is therefore multidisciplinary by necessity, taking into account all available data from clinical symptomatology assessments, histological and electron microscopy tests, and biochemical and molecular genetic studies.

**Table 1:** **Reliability and limitations of diagnostic methods in NPC**

**Diagnostic method**	**Sensitivity**	**Specificity**	**Comment**
Clinical evaluation	Low-to-moderate	Usually low	Depends on the form and stage of disease and experience of clinician
Histology, histochemistry and electron microscopy of appropriate tissues (bone marrow smear, spleen, liver, brain)	Not exactly defined, in particular tissue probably moderate, at autopsy moderate-to-high	High	Must be performed by experienced histopathologist*
Filipin staining test (alone or in combination with LDL-cholesterol assay)	High (but not absolute)	High (but not absolute)	Must be performed by experienced laboratory. Less reliable in persons with ’variant biochemical subtype”. Similar results in some other IEM**.
Molecular genetic analysis	High (but not absolute)	High (but not absolute)	Only one mutation identified in some patients; difficult decision regarding pathogenicity of novel private mutations in NPC genes

*See references [12-15]; **inborn errors of metabolism.

Previous studies in cohorts of selected patients from the UK, Spain, France, and the USA have provided clinical descriptions of NPC [27-30], and have looked into possible genotype–phenotype correlations [24,30-33]. Here, we provide a comprehensive description of a large cohort of NPC patients originating from the area of the contemporary Czech Republic, with an emphasis on how diagnostic procedures have evolved in line with advances in knowledge on the pathogenesis of the disease during the last four decades.

## Materials and methods

### Study design and patients

This study was an observational, retrospective analysis of historic and current information among Czech NPC patients diagnosed between the years 1975 and 2012. All patients with at least one positive, confirmed diagnostic test and with further relevant clinical information available at the Institute of Inherited Metabolic Diseases in Prague were included. This is the only institution that performs diagnostic procedures on lysosomal storage diseases in the Czech Republic.

All available medical reports from identified NPC patients were checked by a clinician familiar with the disease. Relevant clinical data, histopathological and/or ultrastructural findings, biochemical measurements and results of genetic testing were collated. Specific clinical and laboratory findings from siblings or other relatives were also evaluated in relevant cases (e.g. in cases where family members had similar symptoms or identified NPC, prolonged jaundice and/or respiratory failure in the postnatal period).

All information was accessed in accordance with applicable laws and ethical requirements for the study period concerned. All procedures, including informed consents for molecular genetic analyses, were conducted in accordance with the ethical standards of the responsible committee on human experimentation (institutional and national) and the Helsinki Declaration of 1975, as revised in 2000. Informed consent for genetic analysis in some deceased patients was obtained from their legal representatives during genetic counselling for affected families. In cases where genetic information has been included in previous publications, literature citations are provided [33,34].

### Clinical assessments

The types of collated clinical data included information on key signs and symptoms of NPC [1]. Where available, the following were assessed: neurological signs (e.g. oculomotor abnormalities including vertical supranuclear gaze palsy (VSGP), ataxia, dysarthria, dysphagia, dyskinesia, spasticity); neuropsychiatric status (e.g. psychomotor delay and/or regression, learning disabilities, psychosis, behavioural abnormalities, cognitive decline); systemic symptoms (e.g. any evidence of organomegaly, lung disease or neonatal cholestatic disease); relevant imaging findings (e.g. brain MRI, abdominal ultrasonography); results from neurophysiological studies; plasma chitotriosidase activity. As detailed information from MRI and neurophysiological methods was available only in some patients diagnosed in the last decade, these items were not addressed in this analysis. Data from patients treated with miglustat were examined in accordance with defined international guidelines for the diagnosis, assessment and subsequent follow-up of NPC [1,8].

### Histopathological and ultrastructural analyses

#### Bone marrow smears

Bone marrow smears (BMS) were stained by the Giemsa-Romanovsky method and examined for macrophage cytology. Lipopigments and lipids were analysed in an unstained smear mounted in aqueous medium. Autofluorescent lipopigment was detected by epifluorescence microscopy using a fluorescence filter BV-2A (Ex 400–440 nm/DM 455/BA 470) on a Nikon E800 microscope. Lipids were examined for birefringence in polarized light. Activities of non-specific neutral esterases (with α-naphtyl acetate/hexazonium *para*-rosaniline) and acid phosphatase (with naphthol ASBI phosphate/hexazonium *para*-rosaniline) were used for evaluation of the macrophage population and the activated lysosomal system, respectively.

Additional histochemical methods were performed in some cases, including detection of: total phospholipids (with iron hematoxylin); sphingomyelin (iron hematoxylin after alkaline hydrolysis); acidic lipids (with cresyl violet); glycosphingolipids (with periodic acid-Schiff); and apolar components (with Sudan Black B) [14,15].

#### Liver biopsies

Liver biopsy samples were divided into three pieces. One was rapidly frozen for histochemical analyses including detection of phospholipids, sphingomyelin, glycosphingolipids, acidic lipids, and apolar components and lipopigments [13], one was fixed with 4% formaldehyde and embedded in paraffin for routine histology and immunohistochemistry, and one was processed for electron microscopy (see ‘*Electron microscopy*’ section).

#### Brain and visceral organs

Samples of brain and viscera from autopsy examinations were assessed using formol-fixed, paraffin-embedded tissue blocks cut into 4 μm-thick sections and stained using standard histological techniques. Brain samples were processed according to Spilmayer’s scheme, with sections stained using hematoxylin eosin, Nissl, Bodian and Kluver-Barrera methods. A basic set of immunohistochemical methods were applied on paraffin sections of brain for the detection of: glial fibrillary acidic protein (GFAP); microglial marker CD68; neuron-specific nuclear protein (NeuN); neurofilament protein; and ubiquitin. Neurofibrillary tangles were analysed using an anti-phosphorylated tau protein antibody. Membranous (LAMP1, LAMP2) and luminal (cathepsin D) lysosomal markers were assessed to gauge lysosomal storage expansion.

For lipid detection, a battery of histochemical methods was applied on 7–12 μm-thick cryostat sections cut from fresh tissue specimens quenched in petrol ether cooled with a mixture of acetone and dry ice (for brain) or liquid nitrogen. The principal detection methods included: autofluorescence, birefringence, phospholipid detection with iron hematoxylin with or without alkaline pre-hydrolysis, PAS method for detection of glycolipids, cresyl violet for detection of acidic groups, and apolar lipids and lipopigments stained with Sudan Black B [12]. Cholesterol was analysed according to the method of Emeis et al. [12].

#### Electron microscopy

Ultrastructural analysis of neuronal and non-neuronal tissues including skin biopsies (if performed) was conducted using electron microscopy. Tissue samples were fixed with 10% buffered paraformaldehyde and 1% osmium tetraoxide, dehydrated and embedded in Epon-Arraldite mixture. Thin sections were double-contrasted with uranyl acetate and lead nitrate, and examined using a TESLA 500 electron microscope [12].

### Biochemical analyses

Biochemical diagnostic tests in fibroblast cultures were performed based on at least one of three methods referred chronologically depending on the time of technique introduction: 1) measurement of intracellular esterification of exogenous non-lipoprotein ^3^H-cholesterol, expressed as a percentage of total intracellular ^3^H-cholesterol present as cholesteryl esters after 24 hours [17] (two patients); 2) measurement of intracellular esterification of exogenous lipoprotein-derived LDL-cholesterol [18,19]; 3) evaluation of intracellular accumulation of unesterified cholesterol after challenge with lipoprotein-enriched medium, visualized by histochemical staining with filipin [19,20]. Tests 2 and 3 were performed simultaneously at the Laboratoire de Neurochimie, Centre Hospitalier Lyon-Sud in France by Dr M.T. Vanier, MD, PhD.

Final results were interpreted in terms of the ‘classical’, ‘intermediate’ or ‘variant’ biochemical phenotypes. Results supporting a ‘classical’ biochemical phenotype comprised a very strong accumulation of unesterified cholesterol in perinuclear vesicles coupled with a low amount of cholesteryl ^3^H-oleate formed in specific conditions (<100 pmol/mg/4.5 hours; control level 2950 ± 1200 pmol/mg/4.5 hours). A ‘variant’ biochemical subtype was characterized by moderate accumulation of unesterified cholesterol and a relatively high formation of labelled cholesteryl ester (337–1195 pmol/mg/4.5 hours). If the results of both tests were abnormal but there was a discrepancy between filipin staining findings and LDL-cholesterol esterification data, patients were categorized as having an ‘intermediate’ biochemical phenotype.

### Genetic analyses

Genomic DNA was extracted from samples of peripheral blood into EDTA-containing tubes or from skin fibroblast cultures using the QIAamp DNA Blood Mini Kit (Qiagen, Hilden, Germany) according to the manufacturer’s instructions. In the case of previously described adult patient, phenol–chloroform extraction of DNA was used in formalin-fixed, paraffin-embedded (FFPE) tissues as previously described [34]. RNA was extracted from samples of peripheral blood into EDTA-containing tubes or from cultured fibroblasts using the Blood Total RNA Isolation Kit with on-column genomic DNA removal by DNase I (MO-BIO, Carlsbad, CA, USA). Complementary DNA (cDNA) was synthesized using a commercial ThermoScript™ RT reverse transcriptase kit with oligo(dT) (Invitrogen, Carlsbad, CA, USA) according to the manufacturer’s instructions.

Mutation analysis was based on direct sequencing of PCR or RT/PCR products of the *NPC1* (GenBank NC_000018.9; NM_000271.4) and *NPC2* (GenBank NC_000014.8; NM_006432.3) genes using a Pharmacia AlfExpress Sequencer and later capillary sequencers (ABI Prism 3100-Avant or 3500xL Genetic Analyzer; Life Technologies, Carlsbad, CA, USA). The primers used for sequencing were generated in house: their specifications are available upon request. Detected mutations are described according to nomenclature guidelines of the Human Genome Variation Society (HGVS: http://www.hgvs.org/mutnomen/).

The copy number of *NPC1* and *NPC2* coding exons was determined by multiplex ligation probe amplification (MLPA) assay using a SALSA MLPA kit P193-A1 *NPC1* (MRC-Holland, Amsterdam, the Netherlands). The pathogenicity of novel missense variations was evaluated *in silico* using prediction software tools SIFT [35], PolyPhen-2 [36], PMut [37] and MutationTaster [38]. To exclude common polymorphisms, detected gene variations were also compared with databases of the National Center for Biotechnology Information (NCBI; http://www.ncbi.nlm.nih.gov/), Ensembl (http://www.ensembl.org/), 1000 Genomes (www.1000genomes.org/) and the Exome Variant Server (http://evs.gs.washington.edu/EVS/).

### Data analysis

This was an observational study so all data were summarized in a descriptive fashion. No statistical testing was performed. Data on patient and disease characteristics and definitive diagnostic methods were stratified according to established forms of neurological disease in NPC [2,8]. The NP-C SI score was applied retrospectively in 12 patients from whom detailed clinical information was available [26].

## Results

### Patients

A total of 56 patients, 30 (54%) females and 26 (46%) males, were diagnosed with NPC in the Czech Republic between 1975 and 2012. Fifty-five patients were diagnosed with NPC1 and one was diagnosed with NPC2. Overall, 21 patients (38% of the whole cohort) from 10 families were relatives (siblings and cousins). The overall median age of patients at presentation of the first symptom was 5 years, and the median age at diagnostic assessment was 9 years. Patient and disease characteristics data from the whole Czech Republic NPC cohort are summarized in Tables 2,3,4,5,6 according to age at onset of neurological manifestations.

**Table 2:** **NPC patients with neonatal/early-infantile (N/EI) form of disease**

**P**	**G**	**Sb/Cs**	**Year of dg.**	**Age at death (†)/age at last follow up**	**First visceral symptoms (age)**	**First neuro-psychiatric symptoms (age)**	**BMS (Y/ND)**	**Filipin and CEL or CE (Y/ND)**	**Histology and AT (Y/ND)**	**Mutations (** ***NPC1/NPC2†)*** **predicted effect on protein**
			**Age at dg.**							
**Neonatal rapidly fatal form**										
1	M	B of 2/T2	1982	?(†)/1 m	NJa, Hp, HSm (1 m)	NR	*Y*	ND	*Y*	ND
			<1y							
2	M	B of 1/T2	1984	4 m (†)	NJa, Hp, HSm (1 m)	NR	*Y*	ND	*Y (AT)*	ND
			<1y							
3	M	0	1989	2 m (†)	NJa, Hp, HSm (1 m)	NR	ND	ND	*Y (AT)*	c.1261C>T/c.3614C>G††
			<1y							p.Q421*/p.T1205R
**Early infantile form**										
4	F	0	1976	5y (†)	HSm (1y)	PMRt (6 m)	ND	ND	*Y (AT)*	ND
			5y							
5	M	0	1981	5y (†)	NJa, HSm (1 m)	PMRt (1y)	*Y*	ND	*Y (AT)*	c.3557G>A/NDT
			5y							p.R1186H/NDT
6	M	0	1983	4y (†)	NJa, Hp, HSm (1 m)	PMRg (1y)	*Y*	ND	*Y (AT)*	ND
			1y							
7	F	0	1985	11y (†)	mild HSm (1 m)	MRt (1y)	*Y*	*Y (CE)*	*Y*	c.3182T>C/c.3591+1G>A
			1y							p.I1061T/splice
8	F	0	1990	4y (†)	HSm (20 m)	PMRg (20 m)	*Y*	*Y (F/C)*	ND	**c.1812dupT/c.3558delC**
			3y							p.A605Cfs*1/p.A1187Rfs*54
9	F	0	1994	4y (†)	tachypnoea (1 m)	PMRt (1y)	*Y*	*Y (F/C)*	*Y (AT)*	*NPC2*: c.58G>T/c.58G>T
			1y							p.E20*/p.E20*‡
10	F	S of 10/T3	1997	?(†)/5y	NJa, HSm (1 m)	SRt (22 m)	*Y*	*Y (F/C)*	ND	**c.826T>C**/c.3557G>A
			5y							**p.Y276H**/p.R1186H
11	F	0	2001	6y (†)	Sm (2y)	PMRg, A (22 m)	*Y*	*Y (F/C)*	Y (AT)	c.3557G>A/c.3614C>A
			3y							p.R1186H/p.T1205K
12	F	S of 13/T3	2010	6y	HSm (2y)	PMRt, A (18 m)	ND	ND	ND	*c.2196dupT* */c.3557G>A* p.P733Sfs*9 */p.R1186H*
			2y							

*A ataxia, AT autopsy; B brother, BMS bone marrow smear; CE non-LDL-cholesterol esterification test; CEL LDL-cholesterol esterification test; Cs cousins, dg diagnosis, F female, F/C classical biochemical subtype using filipin staining + CEL, G gender, Hp hepatopathy, HSm hepatosplenomegaly, m months, M male, MRt motoric retardation, ND not done, NDT not detected, NR not reported, NJa neonatal jaundice, P order of patient in the age-at-onset category, PMRg psychomotoric regression, PMRt psychomotoric retardation, S sister, Sb sibling, Sm splenomegaly, SRt speech retardation, T table, y years, † patient deceased,* ^†^ *genotype refers to the NPC1 gene (patients 1–8 and 10–12), and to the NPC2 gene (patient 9),* ^‡^ *patient described previously* [33]*,* ^††^ genotype determined indirectly based on parents’ genotype. *translation stop codon in 1-letter amino acid code. Note: novel mutations are highlighted in bold font, and italics indicate decisive diagnostic method or methods (if they were performed simultaneously or in quick succession).

**Table 3:** **NPC patients with late-infantile (LI) form of disease**

**P**	**G**	**Sb/Cs**	**Year of dg**	**Age at death (†)/age at last follow up**	**First visceral symptoms (age)**	**First neuro-psychiatric symptoms (age)**	**BMS (Y/ND)**	**Filipin and CEL or CE (Y/ND)**	**Histology and AT (Y/ND)**	**Mutations (** ***NPC1)*** **Predicted effect on protein**
			**Age at dg**							
1	M	B of 2/T3	1975	11y (†)	NR	PMRt (3 y)	ND	ND	*Y (AT)*	ND
			DPM							
2	M	B of 1/T3	1975	9y (†)	NR	SRt, Epi (3 y)	ND	ND	*Y (AT)*	ND
			DPM							
3	M	0	1975	8y (†)	NR	PMRg, Epi (3 y)	ND	ND	*Y (AT)*	ND
			DPM							
4	F	S of 14/T4	1975	? (†)/4y	HSm (4 m)	PMRg, A (4y)	ND	ND	*Y*	ND
			4y							
5	M	0	1981	5y (†)	HSm (1 m)	PMRt (3y)	Y	ND	Y (AT)	c.1421C>T/c.3557G>A††
			3y							p.P474L/p.R1186H
6	F	0	1988	19y (†)	HSm (2y)	PMRg (6y)	*Y*	*Y (CE)*	*Y*	**c.2635_2636dupAT**/ c.3019C>G
			6y							
										p.Q881Vfs*56/p.P1007A
7	F	0	1990	11y (†)	HSm (4y)	A, Ds, GCt (4 y)	*Y*	*Y (F/V)*	ND	**c.1029dupG**/c.3019C>G
			8y							p.S344Vfs*35/p.P1007A
8	F	0	1993	12 (†) y	NJa (1 m)	A, Ds, Epi (4y)	*Y*	*Y (F/C)*	ND	c.3182T>C/NDT
			10y							p.I1061T/NDT
9	M	0	1995	22y (†)	HSm (5y)	PMRt (5y)	*Y*	*Y (F/I)*	ND	c.3019C>G/c.3557G>A
			5y							p.P1007A/p.R1186H
10	M	B of 10/T2	1998	?(†) /4y	HSm (1 m)	SRt (4 y)	ND	*Y (F/C)*	ND	**c.826T>C**/c.3557G>A
			4y							**p.Y276H**/p.R1186H
11	M	0	2004	10y	HSm (7 m)	A, GCt (4y)	ND	*Y (F/C)*	ND	c.3182T>C/c.3557G>A
			4y							p.I1061T/p.R1186H
12	M	0	2008	12 (†)	Sm (6y)	A, PMRg (3y)	*Y*	ND	ND	*c.3557G>A/c.3557G>A*
			6y							*p.R1186H/p.R1186H*
13	M	B of 12/T2	2009	6y (†)	NJa (1 m)	A, GCt (4y)	*Y*	ND	ND	*c.2196dupT* */c.3557G>A*
			4y							p.P733Sfs*9 */p.R1186H*

*A ataxia, AT autopsy, B brother, BMS bone marrow smear, CE non-LDL-cholesterol esterification test, CEL LDL-cholesterol esterification test, Cs cousin, dg diagnosis, DPM delayed post-mortem examination (in preserved tissues, Ds dysartria, Epi epilepsia, F female, F/C classical biochemical subtype using filipin staining + CEL, F/I intermediate biochemical subtype using filipin staining + CEL, F/V variant biochemical subtype using filipin staining + CEL, G gender, GCt gelastic cataplexy, HSm hepatosplenomegaly, m months, M male, ND not done, NDT not detected, NJa neonatal jaundice, NR not reported, P order of patient at the category; PMRg psychomotoric regression, PMRt psychomotoric retardation, S sister, Sb sibling, Sm splenomegaly, SRt speech retardation, T table, y years, Y Yes, † patient deceased,* ^††^ genotype determined indirectly based on parents’ genotype, *translation stop codon in 1-letter amino acid code. Note: novel mutations are highlighted in bold font, and italics indicate decisive diagnostic method or methods (if they were performed simultaneously or in quick succession).

**Table 4:** **NPC patients with juvenile (J) form of disease**

**P**	**G**	**Sb/Cs**	**Year of dg**	**Age at death (†)/age at last follow up**	**First visceral symptoms (age)**	**First neuro-psychiatric symptoms (age)**	**BMS (Y/ND)**	**Filipin and CEL or CE (Y/ND)**	**Histology and AT (Y/ND)**	**Mutations (** ***NPC1)*** **predicted effect on protein**
			**Age at dg**							
1	F	0	1975	29y (†)	NR	A, Ds (13y)	ND	ND	*Y(AT)*	ND
			29y							
2	M	0	1976	11y (†)	NJa, HSm (1 m)	PMRg (8y)	ND	ND	*Y(AT)*	ND
			11y							
3	F	S of 4/T4	1980	14y (†)	NR	Sp, Epi (7y)	ND	ND	*Y(AT)*	ND
			14y							
4	M	B of 3/T4	1981	20y (†)	Sm (9y)	LDs, Epi (9y)	*Y*	ND	*Y*	ND
			9y							
5	F	0	1981	24y (†)	HSm (8y)	LDs, Tr (8y)	*Y*	ND	*Y*	ND
			10y							
6	F	S of 7/T4	1989	19y (†)	NR	A, PMRg (7y)	*Y*	*Y(F/C)*	*Y*	c.2196dupT/c.2861C>T
			7y							p.P733Sfs*9/p.S954L
7	F	S of 6/T4	1989	33y (†)	HSm (5y)	A, Ds (9y)	*Y*	ND	ND	c.2196dupT/c.2861C>T
			9y							p.P733Sfs*9/p.S954L
8	F	0	1997	24y (†)	NR	Sp, Ds (14y)	ND	ND	Y(AT)	ND
			24y							
9	F	0	1997	18y	HSm (9 m)	A, Ds, BDs (7y)	*Y*	*Y(F/C)*	ND	c.1421C>T/c.2072C>T
			1y							p.P474L/p.P691L
10	F	S of 2/T5	1997	38y	Sm (21y)	A, Ds (13y)	ND	*Y(F/C)*	ND	**c.1210delC**/c.1990G>A
			21y							p.R404Gfs*45/p.V664M
11	M	0	1998	28y	NR	Epi, CDc (13y)	*Y*	*Y(F/V)*	ND	**c.1232G>C**/c.3019C>G
			13y							**p.R411P**/p.P1007A
12	M	Cs of 20/T4	1998	21y	Sm (4y)	Ds (14y)	ND	Y(F/I)	ND	c.2861C>T/c.3557C>A
			5y							p.S954L/p.R1186H
13	M	0	1999	?(†)/17y	Sm (17y)	LDs (7y)	*Y*	*Y(F/I)*	ND	**c.2849T>G**/c.3019C>G
			17y							**p.V950G**/p.P1007A
14	F	S of 4/T3	2000	27y (†)	Sm (7y)	PMRg (7y)	ND	Y(F/V)	Y(AT)	c.352_353delAG/c.3019C>G
			27y							p.Q119Vfs*7/p.P1007A
15	F	0	2000	27y	Sm (13y)	LDs, A (9y)	*Y*	*Y(F/I)*	ND	**c.1812dupT**/c.2861C>T
			13y							p.A605Cfs*1/p.S954L
16	F	0	2002	25y (†)	NR	LDs, A, Dk (13y)	ND	ND	Y(AT)	**c.1028G>A**/c.3019C>G
			25y							**p.G343E**/p.P1007A
17	M	0	2003	35y	Sm (23y)	A, Ds (14y)	*Y*	*Y(F/C)*	ND	c.2861C>T/c.3557G>A
			24y							p.S954L/p.R1186H
18	M	B of 5/T5 &6/T5	2005	23y	Sm (14y)	LDs,Ds (14y)	ND	*Y(F/V)*	ND	*c.2780C>T/c.2780C>T*
			14y							*p.A927V/p.A927V*
19	M	0	2006	27y	Sm (19y)	Ds, Epi (13y)	*Y*	ND	ND	***c.1232G>C*** */c.2861C>T*
			19y							***p.R411P*** */p.S954L*
20	F	Cs of 12/T4	2007	23y	Sm (18y)	Ds, A (13y)	*Y*	ND	ND	*c.2861C>T/c.3557G>A*
			18y							*p.S954L/p.R1186H*

A ataxia, AT autopsy, B brother, BDs behaviour disorder, BMS bone marrow smear, CDc cognitive decline, CE non-LDL-cholesterol esterification test, CEL LDL-cholesterol esterification test, Cs cousin, dg diagnosis, Dk dyskinesia, Ds dysarthria, Epi epilepsia, F female, F/C classical biochemical subtype using filipin + CEL, F/I intermediate biochemical subtype using filipin + CEL, F/V variant biochemical subtype using filipin + CEL, G gender, HSm hepatosplenomegaly, LDs learning disability, m months, M male, ND not done, NJa neonatal jaundice, NR not reported, PMRg psychomotoric regression, P order of patient at the category, S sister, Sb sibling, Sm splenomegaly, Sp spasticity, T table, Tr tremor, y years, Y yes, † patient deceased, *translation stop codon in 1-letter amino acide code. Note: novel mutations are highlighted in bold font, and italics indicate decisive diagnostic method or methods (if they were performed simultaneously or in quick succession).

**Table 5:** **NPC patients with adolescent/adult (A) form of disease**

**P**	**G**	**Sb/Cs**	**Year of dg**	**Age at death (†)/ age at last follow up**	**First visceral symptoms(age)**	**First neuro-psychiatric symptoms (age)**	**BMS (Y/ND)**	**Filipin and CEL or CE (Y/ND)**	**Histology and AT (Y/ND)**	**Mutations (** ***NPC1)*** **predicted effect on protein**
			**Age at dg**							
1	F	0	1980	46y (†)	NR	Tr, CDc, Dk (26y)	ND	ND	*Y (AT)*	ND
			46							
2	M	B of 10/T4	2000	31y (†)	Sm (22y)	CDc, MDs (18y)	*Y*	*Y (F/C)*	ND	**c.1210delC**/c.1990G>A
			23							p.R404Gfs*45/p.V664M
3	M	0	2001	34y (†)	Sm (31y)	SCH (16y)	*Y*	ND	Y (AT)	**c.2712delG**/c.2861C>T
			31							p.Q905Rfs*30/p.S954L
4	F	0	2004	54y (†)	Sm (54y)	NR	ND	ND	*Y (AT)*	**c.1997G>A**/**c.2882A>G**
			54							**p.S666N**/**p.N961S**‡
5	F	S of 6/T5	2005	35y (†)	Sm (27y)	DP (23y)	*Y*	*Y (F/V)*	ND	*c.2780C>T/c.2780C>T*
		& 18/T4	27							*p.A927V/p.A927V*
6	F	S of 5/T5	2005	27y	Sm (20y)	SCH (16y)	ND	*Y (F/V)*	ND	*c.2780C>T/c.2780C>T*
		& 18/T4	20							*p.A927V/p.A927V*
7	F	0	2007	70y	Sm (64y)	A, Ds, Dph (57y)	*Y*	ND	*Y*	*c.2974G>C/c.3182 T>C*
			64							*p.G992R/p.I1061T*
8	F	0	2010	32y	Sm (25y)	A, Dk (16y)	*Y*	ND	ND	***c.1028G>A/c.2198C>G***
			28							***p.G343E*** */* ***p.P733R***
9	F	0	2012	26y	Sm (24y)	LDs (16y)	*Y*	ND	ND	*c.3019C>G/* ***c.3592-7_3592-3delCTTTT***
			24							
										*p.P1007A/splice*

*A ataxia, AT autopsy, B brother, BMS bone marrow smear, CDc cognitive decline, CE non-LDL-cholesterol esterification test, CEL LDL-cholesterol esterification test, Cs cousin, dg diagnosis, Dk dyskinesia, DP depression, Dph dysphagia, Ds dysarthria, F female, F/C classical biochemical subtype using filipin + CEL, F/V variant biochemical subtype using filipin + CEL, G gender, LDs learning disability, M male, MDs memory disorder, ND not done, NR not reported, P order of patient at the age-at-onset category, S sister, Sb sibling, SCH schizophrenia, Sm splenomegaly, T table, Tr tremor, Y yes, y years, † patient deceased,* ^*‡*^ *patient described previously* [34]; *translation stop codon in 1-letter amino acid code. Note: novel mutations are highlighted in bold font, and italics indicate decisive diagnostic method or methods (if they were performed simultaneously or in quick succession).

**Table 6:** **NPC patients with isolated splenomegaly, without neuropsychiatric symptoms**

**P**	**G**	**Sb/Cs**	**Year of dg**	**Age at death (†)/ age at last follow up**	**First visceral symptoms (age)**	**Neuro-psychiatric symptoms (age)**	**BMS (Y/ND)**	**Filipin and CEL or CE (Y/ND)**	**Histology and AT (Y/ND)**	**Mutations (** ***NPC1)*** **predicted effect on protein**
			**Age at dg**							
1	M	0	2009	6y	NJa, Sm (3 m)	NR	*Y*	ND	ND	*c.3557G>A/c.3557G>A*
			1y							*p.R1186H/p.R1186H*
2	M	0	2011	6y	HSm (3y)	NR	ND	ND	ND	*c.2801G>A/c.2861C>T*
			3y							p.R934Q/p.S954L

*AT autopsy, BMS bone marrow smear, CE non-LDL-cholesterol esterification test, CEL LDL-cholesterol esterification test, Cs cousin, dg diagnosis, G gender, HSm hepatosplenomegaly, M male, m months, ND not done, NJa neonatal jaundice, NR not reported, P order of patient at the category, Sm splenomegaly, y years, Y yes. Note: italics indicate decisive diagnostic method or methods (if they were performed simultaneously or in quick succession).*

### Primary and secondary diagnostic tests

**Figure 1:**
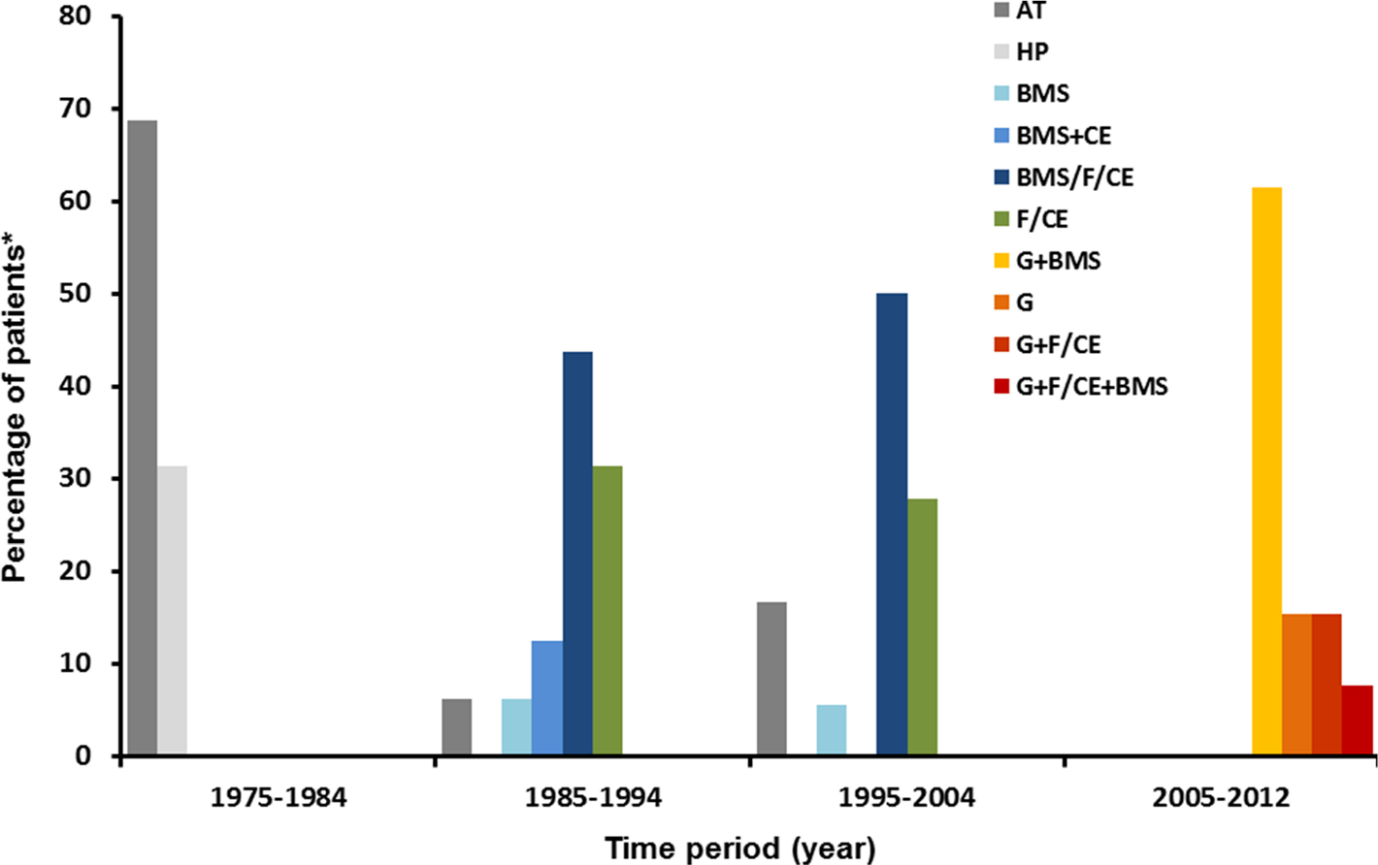
**Definitive diagnostic methods for NPC in the Czech Republic (1975–2012).** *Percentages of patients with each diagnostic pathway calculated relative to total number or patients per time period. AT, autopsy analyses; BMS, bone marrow smear; CE, cholesterol esterification assays; F, filipin test; G, genetic analysis; HP histopathological methods.

Figure 1 summarizes the pathways to confirmed diagnoses in this cohort, stratified by 10-year intervals. Overall, diagnoses were confirmed based on a combination of three methods (histopathological, biochemical, genetic) in 18 patients, two methods in 20 patients, and one method in 18 patients. Twenty-two patients (39%) were diagnosed with NPC primarily based on histopathological information; 16 diagnoses were obtained at autopsy, mainly between 1975 and 1984. A total of 16 patients (29%) were diagnosed based on positive filipin staining results with supportive LDL-cholesterol esterification or on results of a non-LDL-cholesterol esterification test. In both cases, biochemical data were considered in conjunction with suggestive findings from BMS. Five patients were diagnosed on the basis of filipin/LDL-cholesterol esterification tests without preceding BMS information. Eight patients were diagnosed based on genetic and BMS analyses, and two were diagnosed based on genetic analyses alone.

Two pathogenic mutations were detected in the *NPC1* gene in 38 cases from 33 families, and in the *NPC2* gene in one case. NPC diagnoses based on histopathological or biochemical methods were later confirmed by genetic analysis in 26 patients, two of which were established indirectly from parental studies. Two patients (patient 5 in Table 2 and patient 8 in Table 3) were diagnosed by other methods; only one mutation in the *NPC1* gene has so far been detected in these cases.

Median delays between first symptom and diagnosis appeared fairly stable among patients with the neonatal/early infantile form of NPC (1–2 years), but tended to decrease over time in those with the late-infantile form (from 9 years between 1975–1984 down to 3 years over the last two decades). Conversely, diagnostic delays among patients with the juvenile form showed a high degree of variability (average 7 years). Eight patients with the adolescent/adult form were diagnosed in the last two decades (median diagnostic delay 6 years) compared with only one patient from this category in the previous two decades (diagnostic delay 20 years).

### Clinical symptoms

#### Neonatal rapidly fatal form

Three patients had the severe neonatal, rapidly fatal form (Table 2). The first patient suffering from the neonatal form manifested in the first 2 weeks of life with direct hyperbilirubinemia, progressive hepatopathy and prominent hepatosplenomegaly. Liver and bone marrow biopsies were performed at age 2 months with findings supporting a diagnosis of NPC. No other information was available. Direct hyperbilirubinemia was also found in his younger brother at the age of 2 weeks. Prominent hepatomegaly with mild splenomegaly was described shortly afterwards. Surgery for suspected pylorostenosis was performed at age 2 months, with a subsequent revision for volvulus. This patient did not recover after the second operation and died at the age of 4 months. A diagnosis of NPC was based on histopathological findings in his liver, BMS, and examination of autoptic tissues. In the third male, progressive neonatal cholestatic jaundice and hepatosplenomegaly were described. Diagnostic liver biopsy at the age of 6 weeks was complicated by hemoperitoneum, and the patient died several days later. Histopathological examination of autoptic tissues and secondary DNA analysis of the *NPC1* gene confirmed NPC. No neurological symptoms were described in the available reports of any of these three patients.

#### Early-infantile form

Psychomotor retardation or stagnation/regression between 6 months and 2 years of age were the most common symptoms among nine patients with early-infantile onset disease (Table 2). Four patients (patients 5, 6, 7 and 10) had neonatal hepatosplenomegaly and/or prolonged neonatal jaundice. By approximately 2 years of age the leading symptoms were ataxia and speech retardation, after which rapidly progressing neurological manifestations were seen, which included gelastic seizures/cataplexy, oculomotor abnormalities, dysphagia and spasticity. Most of these patients died at approximately 5 years of age due to respiratory complications.

In addition, the only patient with NPC2 (patient 9, Table 2), reported previously [33,39], was included in the early infantile-onset group. This female was born with a delivery complicated by mild asphyxia (Apgar score 3-8-9), and tachypnoea, poor feeding and failure to thrive were observed during the first year of life. Mild psychomotor retardation, central hypotonia and moderate hepatosplenomegaly were described at the age of 1 year, at which point chest X-ray showed lung infiltration and secondary emphysema. Progressive dystrophy, marked hepatosplenomegaly, spasticity and mental decline developed during the second year of life. Lung disease gradually progressed, and at approximately 20 months of age she became oxygen dependent. She died aged nearly 4 years of age due to global respiratory insufficiency.

#### Late-infantile form

Thirteen patients had late infantile-onset NPC (Table 3). The median age at onset of initial symptoms was 2 years. Presenting neurological manifestations included general psychomotor retardation/regression, retardation of speech development, epilepsy, gelastic seizures/cataplexy, ataxia and dysarthria. Four patients (patients 5, 8, 10 and 13, Table 3) had a history of neonatal hepatosplenomegaly and/or prolonged neonatal cholestatic jaundice. VSGP was recorded at an advanced stage of disease only in some of the older reports (together with progressive cognitive and motoric decline), but was recorded more frequently among patients diagnosed in the last decade. Most patients in this age-at-onset subgroup died between 8 and 12 years of age (range 6–19 years), again mostly due to respiratory complications.

#### Juvenile form

Twenty patients had the juvenile-onset form of NPC (Table 4), among whom 10 presented between 7 and 9 years of age with one or more of the following neurological and/or psychiatric signs: psychomotor regression, learning disabilities, behaviour disorder, dysarthria, ataxia, balbuties, epilepsy and spasticity. Ten older patients from this category presented between 13 and 15 years of age with ataxia, dysarthria, cognitive decline, learning disability and epilepsy. Gelastic cataplexy was often present in the younger patients later in the course of the disease. In two cases (patients 9 and 12, Table 4) hepatosplenomegaly or isolated splenomegaly led to the eventual detection of NPC before the appearance of neuropsychiatric manifestations. VSGP and saccadic abnormalities were rarely documented in patients diagnosed before the year 2000, but were often noted in medical reports over the last 10 years. The median age of the first symptoms was 9 years.

Most patients with this age-at-onset form of NPC died in the third decade of their life due to respiratory complications and secondary infection: the oldest living patient is a female aged 38 years (patient 10, Table 4) who scores 18 points on the NPC disability scale [28].

#### Adolescent/adult form

Nine patients had initial neurological or psychiatric signs aged >15 years, and were categorized with the adolescent/adult-onset form of NPC (Table 5). The median age of the first symptoms was 18 years. Some younger patients from this group (patients 2, 8 and 9, Table 5) originally presented with ataxia, dyskinesia, worsening of learning ability and memory disturbances, respectively. These symptoms were followed by VSGP, dysarthria and dysphagia. One female patient (patient 1, Table 5) presented with cognitive deterioration, tremor and dystonia/dyskinesia starting at 26 years of age, with progressive worsening thereafter. Three patients had initial psychiatric diagnoses (two with schizophrenia, one with depression). While two of these cases exhibited subsequent progressive cognitive decline, ataxia, dystonia, dysarthria, dysphagia and VSGP (patients 3 and 5, Table 5), one (patient 6, Table 5) remained free of neurological manifestations at the completion of this study, with only a fluctuating course of psychosis present. One female patient (patient 4, Table 5) died due to a thromboembolism, but the presence of NPC was confirmed based on post-mortem analyses of her liver, spleen and brain [34]. The oldest patient in this cohort (patient 7, Table 5) was diagnosed by chance following examination of her spleen after splenectomy; further analyses revealed that she had been under neurological care for ≥7 years related to spasticity, ataxia, dysarthria and dysphagia, which had previously been ascribed to multisystemic atrophy – she was later found to have VSGP. Overall, four patients from this age-at-onset subgroup are still alive.

### Asymptomatic patients

Two young male patients included in this cohort (Table 6) remained without neurological or psychiatric symptoms throughout the study; both were aged 4 years in the year 2012. These two cases were initially examined due to unexplained splenomegaly (associated with cholestatic neonatal icterus in one case).

### Clinical disease course among related patients

Data on this group of patients are summarized in Table 7. A total of 19 patients were siblings and two were cousins. Two siblings were involved from eight families, and three siblings were from one family. It was not possible to fully assess similarities/differences in some sibling pairs due to incomplete clinical information (mostly in cases diagnosed before the year 2000). A similar course of the disease was reported in siblings from families 1 and 4. Small differences in age at neurological onset were noted in family 5 (sisters with the late infantile- and juvenile-onset forms, respectively), and family 2 (sister and brother with the early infantile- and late infantile-onset forms, respectively) based on subjective reports from parents’ evaluations.

**Table 7:** **Summary of data in related patients in all age-at-onset groups**

**Family number (Sb/Cs)**	**G**	**Age at dg**	**Age at death (†)/age at last follow up**	**First visceral symptoms (age)**	**First euro-psychiatric symptoms (age)**	**Type of primary diagnostic method**	**Mutations in the** ***NPC1*** **gene predicted effect on protein**
FM1 (Sb)	M	1y	? (†)/1y	NJa, Hp, HSm (1 m)	NR	HL, BMS	ND
	M	4 m	4 m (†)	NJa, Hp, HSm (1 m)	NR	BMS, AT	ND
FM2 (Sb)	F	5y	? (†)/5y	NJa, HSm (1 m)	SRt (22 m)	BMS, F(C)	c.826T>C/c.3557G>A
							**p.Y276H**/p.R1186H
	M	4y	? (†)/4y	HSm (1 m)	SRt (4y)	F(C)	c.826T>C/c.3557G>A
							**p.Y276H**/p.R1186H
FM3 (Sb)	M	4y	6y (†)	NJa (1 m)	A, GCt (4y)	BMS, MG	c.2196dupT/c.3557G>A
							p.P733Sfs*9/p.R1186H
	F	2y	6y	HSm (2y)	PMRt (18 m)	MG	c.2196dupT/c.3557G>A
							p.P733Sfs*9/p.R1186H
FM4 (Sb)	M	DPM	11y (†)	NR	PMRt (3y)	AT	ND
	M	DPM	9y (†)	NR	SRt, Epi (3y)	AT	ND
FM5 (Sb)	F	4y	? (†)/4y	HSm (4 m)	PMRg, A (4y)	HL	ND
	F	27y	27 (†)	Sm (7y)	PMRg (7y)	F/V, AT	c.352_353delAG/c.3019C>G
							p.Q119Vfs*7/p.P1007A
FM6 (Sb)	F	14y	14 (†)	NR	Sp, Epi (7y)	AT	ND
	M	9y	20y (†)	Sm (9y)	LDs, Epi (9y)	BMS, HL	ND
FM7 (Sb)	F	7y	19y (†)	NR	PMRg, A (7y)	BMS, HL, F/C	c.2196dupT/c.2861C>T
							p.P733Sfs*9/p.S954L
	F	9y	33y (†)	HSm (5y)	A, Ds (9y)	BMS	c.2196dupT/c.2861C>T
							p.P733Sfs*9/p.S954L
FM8 (Sb)	F	21y	38y	Sm (21y)	A, Ds (13y)	F/C	**c.1210delC**/c.1990G>A
							p.R404Gfs*45/p.V664M
	M	23y	31y (†)	Sm (23y)	CDc, MDs (18y)	BMS, F/C	**c.1210delC**/c.1990G>A
							p.R404Gfs*45/p.V664M
FM9 (Sb)	F	27	35y (†)	Sm (27y)	DP (23y)	BMS, F/V, MG	c.2780C>T/c.2780C>T
							p.A927V/p.A927V
	F	20	27y	Sm (20y)	SCH (16)	F/V, MG	c.2780C>T/c.2780C>T
							p.A927V/p.A927V
	M	14	23y	Sm (14y)	LDs, Ds, (14)	F/V, MG	c.2780C>T/c.2780C>T
							p.A927V/p.A927V
FM10 (Cs)	M	5y	21y	Sm (4y)	Ds, (14y)	F/I	c.2861C>T/c.3557G>A
							p.S954L/p.R1186H
	F	18y	23y	Sm (18y)	Ds, A, (13y)	BMS, MG	c.2861C>T/c.3557G>A
							p.S954L/p.R1186H

A ataxia, AT autopsy, BMS bone marrow smear, CDc cognitive decline, Cs cousins, dg diagnosis, DP depression, DPM delayed post-mortem examination (in preserved tissues), Ds dysarthria, Epi epilepsia, F female, F/C classical biochemical subtype using filipin staining + LDL-cholesterol assay, FM family, F/I intermediate biochemical subtype using filipin staining + LDL-cholesterol assay, F/V variant biochemical subtype using filipin staining + LDL-cholesterol assay, G gender, GCt gelastic cataplexy, HL histology of liver, Hp hepatopathy, HSm hepatosplenomegaly, LDs learning disability, m months, MDs memory disorder, MG molecular genetics, ND not done, NJa neonatal jaundice, NR not reported, PMRg psychomotoric regression, PMRt psychomotoric retardation, Sb siblings, SCH schizophrenia, Sm splenomegaly, Sp spasticity, y years, *translatin stop codon in 1-leter amino acid code. Note: novel mutations are highlighted in bold font.

More precise information was available in family 7 (sisters with juvenile-onset disease) and family 8 (sister with the juvenile-onset form and brother with the adult-onset form). In the first sibling pair the clinical course in the younger sister was clearly milder. In the second pair, initial neuropsychiatric symptoms occurred 5 years later in the brother (at age 18) than in the sister (at age 13). However, disease progression in the brother was more rapid and he died aged 31; his sister (aged 38) is still alive. Siblings from family 3 with the early and late infantile-onset forms showed a 2-year difference in age at neurological onset, which may be associated with more careful monitoring in the younger sister. Disease progression was also slightly milder in the sister, which might be related to her treatment with miglustat over the past years.

Family 9 includes three siblings, one with the juvenile-onset form and two with the adolescent/adult-onset form. As soon as NPC was identified in the oldest sister (presenting with depression, cognitive decline, ataxia and dysarthria aged 27 years), the younger sister and brother were examined. At the time of diagnosis the younger sister had been treated for schizophrenia-like psychosis for 4 years. Ten years after her first presentation of psychosis she had no neurological symptoms. The younger brother had learning difficulties but otherwise his neuropsychiatric status was normal at diagnosis. He remained stable up to 21 years of age, after which he developed sociopathic behaviour, VSGP and ataxia. Miglustat therapy was commenced in all three siblings following its approval in 2011. However, the clinical state of the oldest sister, who was profoundly disabled at the time miglustat therapy was started, continued to worsen rapidly until she died aged 35 years. The younger sister and brother were on miglustat for 1 year and 4 months by the end of the period of observation.

Two patients with the juvenile-onset form from family 10 were first cousins and carried the same *NPC1* gene mutation. The male cousin was diagnosed at 4 years of age because of splenomegaly, and presented with his first neurological symptom (dysarthria) aged 15. Miglustat therapy was commenced in September 2011, and his clinical state appeared stable at the end of the study. In contrast, the female cousin exhibited dysarthria and balbuties from the age of 13 years. Despite commencement of miglustat therapy in August 2011 her clinical state continued to worsen; she currently exhibits epilepsy, VSGP, dysarthria, dysphagia, dystonia, ataxia and cognitive decline.

### NP-C SI

The NP-C SI [26] was applied retrospectively in 12 patients diagnosed during the last 7 years, taking into account the clinical state at the time of diagnosis. Six patients (cases 12 and 13 in Table 3, cases 19 and 20 in Table 4, and cases 8 and 9 in Table 5) scored > 70 points, indicating a high suspicion, four patients (case 12 in Table 2, case 18 in Table 4, and cases 5 and 6 in Table 5) scored 51–55 points, indicating moderate suspicion, and two patients (case 7 in Table 5 and case 1 in Table 6) scored 40 points, indicating borderline suspicion. An asymptomatic patient, case 2 in Table 6, was assessed prospectively, and scored only 20 points on the SI. However, genetic analyses were performed anyway, based on clinical intuition, and led to detection of known pathogenic *NPC1* mutations.

### Histopathological findings

#### Bone marrow

Disease-specific findings based on BMS analyses were recorded in 34 patients in our cohort. Examination of BMS detected foamy histiocytes with variable amounts of autofluorescent ceroid that appeared proportional to patients’ ages and, eventually, in patients with long-term NPC, presented in the form of ‘sea-blue’ histiocytes. NPC histiocytes were distinguishable from those in NPA/B patients based on birefringence of the stored lipids in unstained smears. Lipids in NPC storage cells were always isotropic, but liquid crystals formed in sphingomyelinase deficiency have a high degree of anisotropy, appearing as a Maltese-cross type birefringence in polarized light.

#### Liver biopsy

Findings from combined histological evaluation and histochemical analysis of stored lipids are considered supportive of a diagnosis of NPC. Histopathological examination of liver tissue contributed significantly to NPC diagnoses in four patients in this cohort (case 1 in Table 2, case 4 in Table 3, and cases 4 and 5 in Table 4). Hepatic sphingomyelin storage in NPC was restricted to Kupffer cells; hepatocytes accumulated other types of lipids (cholesterol, glycosphingolipids and phosphoglycerides). Again, this enabled us to distinguish NPC patients from NPA/B patient samples, where both hepatocytes and Kupffer cells accumulate sphingomyelin.

#### Post-mortem analyses

Diagnoses of NPC were based on findings in preserved post-mortem tissue samples in 16 patients. Spleen samples consistently showed signs of storage histiocyte infiltration. Liver changes were minimal, but sometimes showed the presence of foamy Kupffer cells. However, combined histochemical and electron-microscopic examination detected lysosomal storage of mixed lipids in hepatocytes. Adrenal cortex was always unaffected in comparison with NPA/B.

Brain atrophy due to neuronal death was a prominent finding in a number of cases. Widespread lysosomal storage not only in cortical neurons, but also in various subcortical areas (e.g. basal ganglia, cerebellum, brainstem and spinal medulla) was considered characteristic for NPC. Lysosomal storage in brain tissue manifested histologically as a ballooning of neuronal perikarya and frequent storage distension of neurites (meganeurites). Neuroaxonal dystrophy was another typical feature. Neurofibrillary tangles and neuronophagy were demonstrable. Lysosomal storage in central neurons was dominated by accumulation of glycosphingolipids, and thus was different from that seen in histiocytes.

#### Ultrastructural findings

Ultrastructural methods contributed to diagnoses in post-mortem tissue samples as well as in liver biopsies. However, no patients of this cohort were diagnosed primarily on the basis of ultrastructural skin examination. Lysosomal ultrastructure in all affected cell types was lamellar, loose and concentric, and was arranged as rings, vesicles or with a larger annular formation. These structures were described as polymorphous cytoplasmic bodies, oligomembranous bodies or multivesicular bodies. Vesicular and multivesicular structures appeared specific for NPC.

### Biochemical phenotype

Filipin staining and simultaneous LDL-cholesterol esterification tests were performed in a total of 22 patients. Non-LDL-cholesterol esterification tests were performed in two patients diagnosed in 1985 and 1988. In all cases, results of biochemical testing were supportive of NPC diagnoses. Overall, based on both filipin staining and LDL-cholesterol esterification tests, 12 patients (four with the neonatal/early-infantile form, three with the late-infantile form, four with the juvenile form and one with the adult-onset form) were shown to have a ‘classical’ biochemical phenotype, four patients (one with the late-infantile form and three with the juvenile-onset form) had an ‘intermediate’ biochemical phenotype, and six patients (one with the late-infantile form, three with the juvenile form and two with the adult-onset form) had a ‘variant’ biochemical phenotype.

### Genetic analysis

*NPC1* genotyping analyses indicated pathological mutations in 38 patients from 33 families. *NPC2* mutant genotyping was positive in one patient (patient 9 in Table 2). In total, 30 different mutations were detected, 14 of which were confirmed as being novel. Twenty out of 30 different mutations were family specific and 10 were recurrent. Interestingly, among the recurrent mutations there were three that were not described previously in the literature (p.G343E, p.R411P and c.1812dupT). Each of these novel mutations occurred in two unrelated probands in our cohort.

Counting each mutated allele in each family only once, the most frequent mutant *NPC1* allele was p.R1186H (n = 13), followed by p.P1007A (n = 8), p.S954L (n = 8) and p.I1061T (n = 4). Using both sequencing analysis and MLPA, only one pathogenic mutation was found in heterozygous form in two patients diagnosed by other methods: p.R1186H in patient 5, Table 2 and p.I1061T in patient 8, Table 3. Both of these patients had infantile-onset NPC and presented with a ‘classical’ biochemical phenotype. *NPC1* genotypes were determined indirectly based on parental genetic analyses in two patients who were diagnosed based primarily on histopathological findings (patient 3 in Table 2 and patient 5 in Table 3).

### Prenatal diagnostics

Although genetic counselling was offered and carried out in most of the affected families, prenatal diagnostics for 25% risk of NPC were conducted in only four women from four families. Filipin staining in combination with LDL-cholesterol esterification assay were performed in cultivated chorionic villi cells from two foetuses. In the both cases, results testified to a healthy foetus which was subsequently confirmed by DNA analysis in the concerned siblings. After 2005, prenatal testing through targeted DNA analysis of the familiar *NPC1* mutations in chorionic villi cells was performed in two foetuses who were both shown to be healthy heterozygotes.

### Chitotriosidase activity

Plasma chitotriosidase activity has been measured as a marker of reticuloendothelial activation at our centre since 2001, with a reference range of 4.4–89 nmol/h/ml. Overall, chitotriosidase activity was measured in 20 patients; the median value across the cohort was 195 nmol/h/ml. Chitotriosidase was measured in 14 patients at the time of diagnosis (one early-infantile, two late-infantile, four juvenile, and five adult-onset patients as well as two pre-symptomatic cases); results ranged from 39 nmol/h/ml to 793 nmol/h/ml. Recorded values were in the normal range in two of the juvenile-onset patients (cases 19 and 20 in Table 4) and two of the adult-onset patients (cases 8 and 9 in Table 5). Elevated values were recorded in the remaining 16 patients.

## Discussion

Data from this observational study in a large cohort of Czech NPC patients clearly demonstrate the evolution of the diagnostic process in NPC over the last four decades, stemming from the rapid adoption of new clinical methods based on research activities. We also highlight the crucial role of close collaboration between treating physicians and specialized diagnostic laboratories and reference centres in a rare disease like NPC.

Up until 1985 almost all NPC patients, including those from our centre, were diagnosed exclusively on the basis of histopathological evidence, often during post-mortem studies. Preserved tissues were even stored for several years until attained knowledge allowed diagnosis in three late-infantile cases. Histological, histochemical and ultrastructural features allowing the efficient establishment of diagnoses at the clinico-pathological level were summarized by Elleder in 1989 [40]. Liver findings in seven patients from this cohort who had neonatal, early-infantile, late-infantile or juvenile-onset disease were published in detail in 1984 [13], and an in-depth neuropathological study of two patients from this cohort who had the early-infantile onset form was published in 1985 [12].

The introduction of the filipin staining test and cholesterol esterification assays into clinical practice around 1985 led to a vast improvement in the diagnosis of NPC, particularly among younger-onset patients. However, these biochemical methods came with the limitations of being both labour intensive and time-consuming, and necessitated skin biopsy and fibroblast cultivation. In addition, false-positive diagnoses were seen, and the reliability of findings was lower among patients with ‘variant’ biochemical phenotypes. As biochemical tests required referral to a specialist laboratory, information from histopathological BMS analyses (e.g. findings of isotropic histiocytes based on birefringence) proved invaluable in our institution for selecting patients with a high likelihood of having NPC for further diagnostic work-up [14]. A histochemical study of lipid storage in BMS, which included findings from five patients from this cohort with infantile or childhood-onset NPC, was published by Elleder in 1983 [15].

Molecular genetic analyses can be performed in DNA or RNA isolated from blood samples, and are more suitable for the purposes of genetic counselling and prenatal diagnostics. Improvements in the accessibility and accuracy of genetic profiling together with an increased understanding of the clinical symptomatology and natural history of NPC have enabled the earlier detection of the disease in the Czech Republic, especially among patients with the adolescent/adult-onset form. In 2010, Poupetova et al. [4] reported that the birth prevalence of NPC was 0.91 per 100,000 (originally 0.66–0.83 per 100,000 in the UK, France and Germany). Taking our data into account, the revised estimate for the birth prevalence for NPC for the period 1975–2012 is assessed at 0.93 per 100,000. The detection rate for the Czech Republic population, which has approximately 10 million inhabitants, is estimated at 1 to 2 patients per year [4].

Reported data on presenting clinical symptoms and natural course of the disease in the Czech NPC patients are comparable with the data from other large cohorts [7,10,27-29]. Our experience that subtle, unspecific symptoms like learning disabilities, stuttering, clumsiness, fatigue and mood disorder often precede more specific manifestations of the disease by many years is in accordance with results of clinical database analysis in a large group of German and Swiss patients [41].

Notably, a total of 14 novel mutations have been identified by genetic analysis at our laboratory since 2005. While the pathogenicity and novelty of these missense mutations has been confirmed by various *in silico* methods, and by cross reference with large mutation databases, respectively, they are not yet listed in the *NPC1* gene mutations database.

It is interesting to note that the frequency of individual mutated *NPC1* alleles in our cohort differs compared with previous published information. While data indicate that p.I1061T is the most frequent mutation in patients of Western European descent, accounting for approximately 20% of recorded alleles in the UK and France [1], it was present in only four alleles (6%) in our cohort. The second most frequent mutation in Europe, p.P1007A, was present in eight alleles (12%) among our patients. The same frequency was found for p.S954L, which is also common in European patients. The most frequent *NPC1* mutation in our cohort was p.R1186H (20%). A founder effect for this mutation is considered unlikely as it is associated with at least two different haplotypes (data not shown).

The availability of both clinical data and genetic information concerning *NPC1* mutations in this cohort allowed some appraisal of a number of genotype–phenotype correlations. Mutation p.R1186H was present in homozygous state in a patient with late infantile-onset NPC (patient 12 in Table 3) and in the 4-year-old asymptomatic patient (patient 1 in Table 6) who has been on miglustat therapy since 2011. This mutation was also detected in combination with other missense or frame shift mutations in nine patients with infantile forms of the disease. Three patients with juvenile-onset disease were compound heterozygotes (p.R1186H/p.S954L); the p.S954L mutation is not present in infantile-onset forms of NPC, but was present in seven patients with the juvenile-onset form (one being neurologically asymptomatic up to 15 years of age) and one adult-onset case. Based on these data, the p.R1186H mutation can be considered as a severe mutation. In contrast, p.S954L may attenuate phenotypic manifestations, as it occurred in combination with severe mutations such as deletions or duplications causing frame-shift mutations in patients with milder forms of disease (juvenile-onset patients 6, 7 and 15 in Table 4 and adult-onset patient 3 in Table 5).

Mutation p.P1007A was shown to occur frequently among patients with late-infantile or juvenile-onset NPC, indicating a possible moderate effect of the mutation on patient phenotype. In one adult-onset patient (patient 9 in Table 5), the mutation was present in combination with an intronic deletion (c.3592-7_3592-3delCTTTT) that leads to a transcriptional splicing error resulting in the deletion of mRNA exon 24. However, the presence of erroneously as well as correctly spliced transcript cannot be excluded, and the minor fraction of a correctly spliced transcript may lead to a milder phenotype.

The most frequent *NPC1* mutation in Western Europe and the US, p.I1061T [27-32], associated mostly with the juvenile-onset form of the disease, was detected in the Czech cohort in one patient with the early infantile form (patient 7, Table 2), two patients with the late infantile form (patients 8 and 11, Table 3) and in the oldest adult NPC patient (patient 7, Table 5). In the first case, the p.I1061T was found in heterozygous form with a likely deleterious splice mutation, in another patient with late infantile-onset NPC it was in heterozygous form along with the very likely severe mutation p.R1186H, mentioned above, and in the other late infantile-onset patient, diagnosed biochemically, the second mutation has not yet been found. These results support the opinion that the severe phenotype in our infantile patients was not conditioned by the p.I1061T mutation, but by the second one. The adult patient was a compound heterozygote for the p.I1061T and p.G992R mutations. The p.G992R in homozygous state has previously been described in another patient with a non-neuronopathic adult-onset form of NPC [42]. Together, these findings are in accordance with the assumed moderate effect of the referred mutation.

A structured approach to the diagnosis of NPC has been developed in our centre. If results from a range of clinical evaluations (assessment of family and personal history, examination by a clinician familiar with NPC, abdominal ultrasonography and plasma chitotriosidase activity) support suspicion of NPC, we usually recommend bone marrow biopsy. Genetic analyses of NPC genes are usually undertaken if specific BMS histiocytes are found or if a clinical suspicion of NPC persists after meticulous re-evaluation (with filipin testing) in the absence of specific histiocytes. Novel, emerging diagnostic methods (plasma oxysterols and/or sphingolipid profiling, next-generation sequencing panels) alongside scoring systems such as the NP-C SI may further enhance this diagnostic algorithm in the coming years.

## Conclusion

Relevant clinical evaluations combined with histopathological, biochemical and genetic analyses have contributed to an effective diagnostic process for NPC in the Czech Republic that has resulted in a surprisingly stable rate of diagnosis over four decades. The only exception is the adolescent/adult-onset subgroup, where there has been a clear proportional rise in the number of cases confirmed during the last 15 years.

Most patients diagnosed in recent years can gain access to miglustat therapy, and treatment with this agent has been commenced in 18 patients at our centre subsequent to its approval in August 2011. The rational use of improved techniques for detecting and diagnosing NPC may allow the earlier initiation of disease-specific therapy. Based on international recommendations for the clinical management of NPC it is hoped that this may impact positively on long-term treatment outcomes [1,8].
